# Early Donor-Derived Cell-Free DNA Kinetics After Kidney Transplantation in Atypical Hemolytic Uremic Syndrome: A Preliminary Observational Study

**DOI:** 10.3390/ijms27167331

**Published:** 2026-08-17

**Authors:** Patryk Wawrzonkowski, Kornelia Gajek, Weronika Smulska, Katarzyna Grabka, Karolina Marek Bukowiec, Magdalena Kuriata-Kordek, Patryk Jerzak, Adrian Domagalski, Marek Ussowicz, Mirosław Banasik

**Affiliations:** 1 Department of Nephrology, Transplantation Medicine and Internal Diseases, Institute of Internal Diseases, Wroclaw Medical University, 50-551 Wroclaw, Poland; w.smulska@gmail.com (W.S.); anbek96@gmail.com (K.G.); karolina.marek-bukowiec@umw.edu.pl (K.M.B.); magdalena.kuriata-kordek@umw.edu.pl (M.K.-K.); patryk.jerzak@umw.edu.pl (P.J.); adrian.domagalski0@gmail.com (A.D.); 2 University Clinical Hospital in Wroclaw, Borowska 213, 50-556 Wroclaw, Poland; 3 Department of Pediatric Bone Marrow Transplantation, Oncology and Hematology, Wroclaw Medical University, 50-551 Wroclaw, Poland; kornelia.gajek@umw.edu.pl (K.G.); ussowicz@tlen.pl (M.U.)

**Keywords:** donor-derived cell-free DNA, dd-cfDNA, atypical hemolytic uremic syndrome, aHUS, kidney transplantation, thrombotic microangiopathy, complement, rejection, donor-specific antibodies

## Abstract

Donor-derived cell-free DNA (dd-cfDNA) is an emerging non-invasive biomarker of kidney allograft injury. Elevated dd-cfDNA has been associated with acute rejection, particularly antibody-mediated rejection; however, dd-cfDNA reflects graft-derived tissue injury rather than a specific alloimmune mechanism. Its interpretation may therefore be challenging in conditions characterized by endothelial and microvascular injury, such as atypical hemolytic uremic syndrome (aHUS). This retrospective observational study included 14 kidney transplant recipients who underwent their first kidney transplantation at Wroclaw Medical University between April and December 2022. Twelve recipients without aHUS and two recipients with aHUS were assessed at day 7 (D7), day 14 (D14), month 1 (M1), and month 2 (M2). Protocol biopsies were not systematically performed. In recipients without aHUS, median %dd-cfDNA declined from 0.72% (range, 0.26–2.33%) at D7 to 0.12% (range, 0.05–0.34%) at M2. In the two recipients with aHUS, values remained near or above 1% through M1 and declined at M2. No donor-specific antibodies against human leukocyte antigens (HLA) or clinically documented rejection occurred. Exploratory analyses did not identify a statistically significant association between %dd-cfDNA and serum creatinine (Spearman rho = 0.09, *p* = 0.541). These recipient-level observations suggest that early dd-cfDNA kinetics in the two recipients with aHUS differed from the declining pattern in the small comparison cohort, but they do not establish a group-level difference. Because protocol biopsies were not systematically performed, subclinical rejection could not be excluded. The findings should be considered hypothesis-generating and support cautious interpretation of universal dd-cfDNA thresholds in aHUS.

## 1. Introduction

### 1.1. Donor-Derived Cell-Free DNA: A Promising Non-Invasive Biomarker

Kidney transplantation remains the preferred treatment for patients with end-stage renal disease, offering improved survival and quality of life compared with dialysis [1]. Despite advances in immunosuppression and post-transplant care, allograft rejection continues to threaten long-term graft survival and function. Acute rejection may lead to irreversible graft injury if not identified and treated promptly [2].

The current diagnostic reference standard for rejection is histopathological evaluation of kidney allograft biopsy specimens. Although biopsies provide essential diagnostic information, they are invasive, carry procedural risks such as bleeding or infection, and may be affected by sampling error and interobserver variability [3,4]. These limitations have stimulated the search for safer, repeatable, and more objective diagnostic adjuncts.

One of the most promising approaches is donor-derived cell-free DNA (dd-cfDNA), a fragment of DNA released into the recipient circulation as a result of allograft cell injury and turnover. Cell-free DNA (cfDNA) is generated predominantly during apoptosis and necrosis, and its presence in circulation may reflect tissue injury. In transplant recipients, the proportion of cfDNA originating from donor tissue provides a quantitative measure of allograft-derived injury [5].

Numerous studies have demonstrated an association between elevated dd-cfDNA and episodes of acute rejection, with increased levels indicating heightened cell turnover and injury in the transplanted organ [6,7]. Before detecting changes in conventional clinical markers like serum creatinine or proteinuria, dd-cfDNA levels may rise, potentially allowing for earlier clinical intervention [8]. This has positioned dd-cfDNA as a useful adjunct in post-transplant surveillance and in the assessment of immunological risk [9].

Beyond rejection, dd-cfDNA has also shown promise in identifying other forms of graft injury, including BK virus nephropathy and ischemia–reperfusion injury, although specificity remains a major concern [10,11,12]. Overall, dd-cfDNA offers an appealing, non-invasive tool for post-transplant monitoring, yet important diagnostic caveats remain, particularly in recipients with complex comorbidities or rare conditions such as atypical hemolytic uremic syndrome (aHUS).

### 1.2. Diagnostic Challenges of dd-cfDNA in Kidney Transplant Recipients with aHUS

Atypical hemolytic uremic syndrome is a rare, life-threatening disease characterized by complement dysregulation leading to thrombotic microangiopathy, endothelial damage, and multiorgan injury, with the kidneys being among the most commonly affected organs [13,14]. Patients with aHUS frequently progress to end-stage renal disease and may require kidney transplantation. However, transplant outcomes in this population remain complex because of the risk of disease recurrence and persistent microvascular injury after transplantation [15].

These pathophysiological mechanisms pose a unique challenge for the interpretation of dd-cfDNA. Chronic, recurrent, or subclinical endothelial injury associated with aHUS, even in the absence of overt alloimmune activation, could plausibly contribute to graft-derived cell injury and dd-cfDNA release. This raises concerns about the specificity of dd-cfDNA as a marker of rejection in this context, because elevated values may not necessarily reflect immune-mediated allograft injury but could instead reflect complement-mediated endothelial injury and associated cell turnover [16]. Although complement-mediated endothelial injury is an established feature of aHUS and dd-cfDNA reflects allograft-derived injury, a direct association between complement-mediated endothelial injury and sustained dd-cfDNA elevation has not been established in kidney transplant recipients with aHUS. The present study therefore explored this potential relationship as a hypothesis rather than testing a confirmed mechanistic association.

Previous studies have shown that the interpretation of dd-cfDNA depends not only on its absolute value but also on its temporal pattern. In general kidney transplant populations, persistently elevated dd-cfDNA has been associated with subsequent development of de novo donor-specific antibodies and deterioration of graft function [17]. Elevated dd-cfDNA may also precede biopsy-proven rejection [18], while persistent values above commonly applied thresholds during surveillance have been linked to non-improving graft function [19]. Moreover, increased dd-cfDNA has been reported in BK polyomavirus-associated nephropathy [10,11] and during early post-transplant graft injury [12]. However, none of these studies specifically characterized early dd-cfDNA kinetics in kidney transplant recipients with aHUS. Therefore, the clinical significance of persistent dd-cfDNA elevation in this population remains uncertain. This knowledge gap provided the rationale for the present preliminary study.

### 1.3. Aim of the Study

This preliminary observational study sought to address this knowledge gap by describing the early dynamics of plasma dd-cfDNA after kidney transplantation in recipients with and without aHUS. By evaluating dd-cfDNA in relation to donor- specific antibody (DSA) status, serum creatinine, and the documented clinical course, the study aimed to explore potential limitations in the interpretation of dd-cfDNA as a biomarker of graft injury in the specific context of aHUS, rather than to establish its diagnostic reliability.

## 2. Results

Recipient-level demographic and peri-transplant characteristics, including age and sex, are presented in Table 1. Non-aHUS and aHUS recipients are shown as separate patient-level rows.

Overall, maintenance immunosuppression was based on corticosteroids, tacrolimus, and mycophenolate mofetil in all recipients. Delayed graft function was recorded in one non-aHUS recipient and in one aHUS recipient. Cohort characteristics and the testing schedule are summarized in Table 2.

**Table 2:** Cohort characteristics and testing schedule in kidney transplant recipients with and without aHUS.

Variable	Non-aHUS Recipients	aHUS Recipients
Number of recipients	12	2
First kidney transplantation	12/12	2/2
Study period	April–December 2022	April–December 2022
Plasma collection time points	D7, D14, M1, M2	D7, D14, M1, M2
Clinical graft status during observation	Stable renal function during follow-up	Preserved graft function during follow-up
Clinically documented acute rejection	None documented	None documented
Protocol biopsies	Not performed systematically	Not performed systematically
DSA detected during follow-up	0/12	0/2
dd-cfDNA assay	AlloSeq cfDNA, NGS	AlloSeq cfDNA, NGS
DSA assay	LABScreen Single Antigen Beads	LABScreen Single Antigen Beads

Abbreviations: aHUS, atypical hemolytic uremic syndrome; cfDNA, cell-free DNA; D7, day 7; D14, day 14; dd-cfDNA, donor-derived cell-free DNA; DSA, donor-specific antibody; M1, month 1; M2, month 2; NGS, next-generation sequencing. Available dd-cfDNA measurements in recipients without aHUS were n = 11 at day 7 (D7), n = 9 at day 14 (D14), n = 11 at month 1 (M1), and n = 10 at month 2 (M2); both recipients with aHUS had measurements at all four time points. In recipients without aHUS, median %dd-cfDNA was 0.72% (range, 0.26–2.33%) at D7, 0.62% (range, 0.23–1.85%) at D14, 0.26% (range, 0.10–1.07%) at M1, and 0.12% (range, 0.05–0.34%) at M2. In recipient A1, dd-cfDNA values were 1.06%, 1.58%, 1.14%, and 0.30% at D7, D14, M1, and M2, respectively; corresponding values in recipient A2 were 1.14%, 1.07%, 0.88%, and 0.40% (Figure 1).

**Figure 1:**
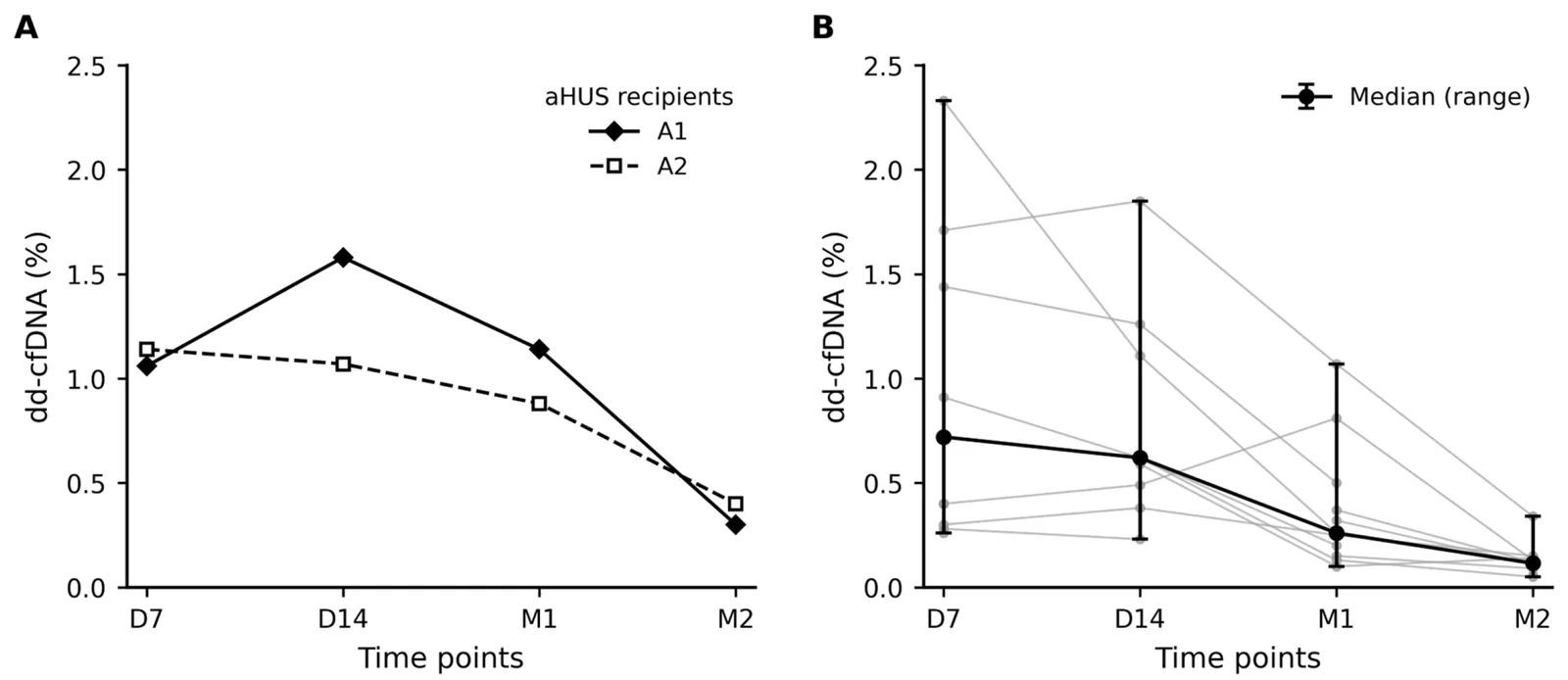
Individual early post-transplant dd-cfDNA trajectories in kidney transplant recipients with and without atypical hemolytic uremic syndrome (aHUS). (**A**) Separate trajectories for recipients A1 and A2 with aHUS. (**B**) Individual trajectories for recipients without aHUS are shown as thin lines with open circles; the black line and filled circles represent the median, and dispersion bars represent the full observed range. The thin gray lines represent individual longitudinal dd-cfDNA trajectories of non-aHUS recipients. Abbreviations: dd-cfDNA, donor-derived cell-free DNA; D7, day 7; D14, day 14; M1, month 1; M2, month 2.

**Figure 2:**
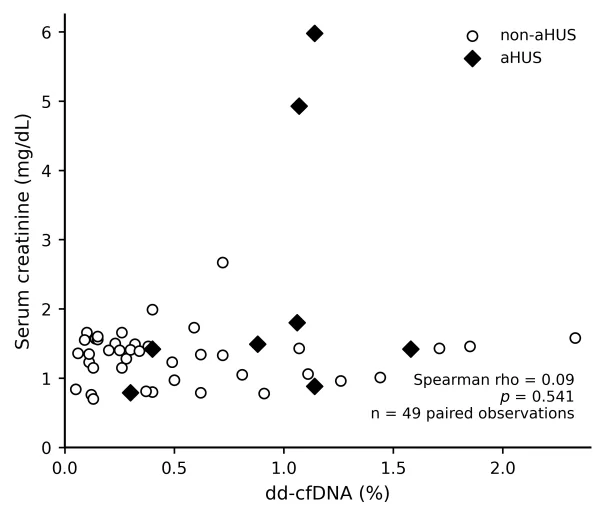
Exploratory relationship between %dd-cfDNA and serum creatinine in 14 kidney transplant recipients across D7, D14, M1, and M2. Open circles represent observations from recipients without aHUS, and filled diamonds represent observations from the two recipients with aHUS. The plot includes 49 paired observations. The Spearman correlation was not statistically significant (rho = 0.09, *p* = 0.541). Repeated observations from the same recipients are displayed and should not be interpreted as independent measurements. Abbreviations: aHUS, atypical hemolytic uremic syndrome; dd-cfDNA, donor-derived cell-free DNA; D7, day 7; D14, day 14; M1, month 1; M2, month 2; mg/dL, milligrams per deciliter.

No donor-specific anti-HLA antibodies were detected in any recipient during the observation period, and no clinically documented acute rejection occurred. In an exploratory analysis including all 14 recipients and the four prespecified post-transplant time points, no statistically significant association was detected between %dd-cfDNA and serum creatinine (Spearman rho = 0.09, *p* = 0.541; 49 paired observations; Figure 2). A partial Kendall correlation controlling for sampling time was also negligible (tau = −0.03, *p* = 0.786).

## 3. Discussion

This study examined early dd-cfDNA kinetics in kidney transplant recipients with and without aHUS. In the non-aHUS comparison cohort, dd-cfDNA declined over the early post-transplant period. In the two recipients with aHUS, values remained near or above 1% through M1 and then declined at M2, despite the absence of detectable DSA, clinically documented rejection, and deterioration of graft function during follow-up. These recipient-level observations should not be interpreted as establishing a pattern applicable to the broader aHUS population.

These findings raise important questions regarding the interpretation of dd-cfDNA in the context of aHUS. Previous studies in broader kidney transplant populations have consistently shown that dd-cfDNA values around or above 1% are associated with acute rejection and poorer graft outcomes [6,7,8]. In the two recipients described here, early values near or above this threshold may not have carried the same clinical meaning as in unselected kidney transplant populations. One biologically plausible, but unconfirmed, explanation is that the observed values could partly reflect endothelial injury and cell turnover related to complement-mediated pathology rather than exclusively alloimmune-mediated graft damage [10,11,12,13]. However, the present data do not establish this mechanism, and alternative explanations, particularly subclinical rejection, remain possible. These observations therefore raise a hypothesis rather than demonstrating that aHUS has a disease-specific dd-cfDNA profile.

The role of dd-cfDNA as a biomarker of allograft rejection has been progressively refined since its initial clinical validation [5,20]. A 1% threshold discriminates active rejection with high negative but limited positive predictive value, reflecting that dd-cfDNA rises with any source of graft injury rather than with rejection specifically [5]. This signal is strongest for antibody-mediated rejection, in which microvascular inflammation produces greater endothelial DNA release than the predominantly tubulointerstitial injury of T-cell-mediated rejection [9,21]. A recent meta-analysis confirmed higher pooled diagnostic accuracy for antibody-mediated than for any-cause rejection [8]. Even low-grade or borderline T-cell-mediated rejection may, however, be accompanied by elevated dd-cfDNA and an increased risk of subsequent graft injury [22], underscoring that the biomarker reports tissue injury along a continuum rather than a binary rejection state.

Beyond a single cross-sectional measurement, the temporal behavior of dd-cfDNA carries additional prognostic information. In the longitudinal Assessing Donor-Derived Cell-Free DNA Monitoring Insights of Kidney Allografts with Longitudinal Surveillance (ADMIRAL) cohort, persistently elevated values, rather than isolated peaks, predicted future de novo donor-specific antibodies and a clinically meaningful decline in glomerular filtration rate, and elevations may precede biopsy-proven rejection by several weeks [17,18]. Surveillance studies using the same next-generation sequencing assay applied here have similarly linked persistent early post-transplant elevation to non-improving graft function [19]. This kinetic perspective is relevant to the present cohort: a transient perioperative rise that resolves is expected, whereas values that remain elevated may warrant closer evaluation. In the two recipients with aHUS described here, complement-mediated endothelial injury represents one possible mechanism that could have contributed to continued dd-cfDNA release; however, this mechanism was not directly assessed and cannot be confirmed by the present data.

The lack of published information on dd-cfDNA changes in aHUS makes interpretation difficult. It remains unclear whether the biomarker behaves similarly to other transplant populations or whether early values in aHUS reflect perioperative injury, baseline vascular injury, recurrent or subclinical thrombotic microangiopathy, subclinical rejection, or overlapping mechanisms. This knowledge gap calls for studies specifically addressing rare transplant populations, because a fractional threshold alone cannot distinguish among these processes.

None of the recipients developed detectable DSA during follow-up, and the exploratory analyses did not identify a statistically significant association between %dd-cfDNA and serum creatinine. The limited sample size resulted in limited statistical power, particularly for detecting weak or moderate associations. Therefore, the absence of a statistically significant association between %dd-cfDNA and serum creatinine should not be interpreted as evidence of no association. Serum creatinine is an insensitive and delayed marker of injury, whereas dd-cfDNA may reflect biological processes not captured by routine biochemical testing. Importantly, recent evidence shows that antibody-mediated rejection can occur even in the absence of detectable DSA, with DSA-negative antibody-mediated rejection sharing molecular signatures and dd-cfDNA release patterns with DSA-positive cases [23]. Therefore, a negative DSA result and stable creatinine do not fully exclude alloimmune injury.

In the two recipients with aHUS described here, early dd-cfDNA values near or above 1% in the absence of detectable DSA could potentially reflect non-alloimmune endothelial injury, low-grade immune activation below the threshold of serological detection, subclinical rejection, or a combination of these processes. The present study cannot distinguish among these explanations. Accordingly, dd-cfDNA should not be interpreted as a stand-alone diagnostic test in this setting. A multimodal approach integrating DSA, graft function, proteinuria, infection status, hemolysis and thrombotic microangiopathy markers, complement activation markers, and biopsy findings when clinically indicated is likely to provide a more reliable assessment.

Our study adds preliminary recipient-level observations to the limited literature addressing dd-cfDNA kinetics in rare transplant populations. The values observed in the two recipients with aHUS highlight the need to investigate whether interpretation of this biomarker requires additional clinical context in the presence of complement-mediated microvascular disease. These observations do not establish a disease-specific dd-cfDNA pattern or justify a separate threshold for the broader aHUS population. Future studies should integrate dd-cfDNA with complement activation products, endothelial injury markers, histopathology, and molecular graft assessment to help distinguish alloimmune from non-alloimmune injury.

This study has important limitations. First, only two recipients with aHUS were included; therefore, the findings are hypothesis-generating and do not support group-level inference. Second, the study was retrospective and single-centre, and no formal a priori sample-size calculation was performed because of its exploratory design and the rarity of aHUS. Third, the groups were not matched for demographic and peri-transplant characteristics. Differences in age, sex, cold ischemia time, delayed graft function, post-transplant dialysis, and induction therapy may have influenced early dd-cfDNA kinetics independently of aHUS. Sex could not be evaluated as an independent biological variable because both recipients with aHUS were female and the cohort was too small for sex-stratified analysis. Fourth, protocol biopsies and molecular biopsy assessment were not systematically available, so subclinical rejection could not be excluded. Fifth, detailed markers of complement activation and thrombotic microangiopathy were not available for integrated analysis. Sixth, not all recipients had paired dd-cfDNA and serum creatinine measurements at every prespecified time point; the exploratory correlation analysis therefore included 49 paired observations and remained limited by repeated measurements within recipients. Seventh, dd-cfDNA was analysed as a fractional value; absolute quantification may provide additional information. Furthermore, the absence of an additional external reference group limits the ability to distinguish a potential effect of aHUS from other recipient- and transplant-related factors. Finally, follow-up was limited to the early post-transplant period.

## 4. Materials and Methods

This retrospective observational study included 14 kidney transplant recipients: 12 recipients without aHUS and 2 recipients with aHUS. Recipient-level age, sex, and peri-transplant characteristics are reported in Table 1. The aHUS subgroup consisted of two female recipients aged 39 and 22 years. Both received a kidney from a deceased donor. In one recipient, aHUS was the primary cause of kidney failure, whereas in the other, aHUS was considered most likely secondary to primary focal segmental glomerulosclerosis.

The non-aHUS comparison group constituted a convenience sample of kidney transplant recipients from the same transplantation period for whom stored plasma samples suitable for dd-cfDNA analysis were available in the study database. No matching procedure was applied.

In the first patient, genetic testing performed as part of the diagnostic work-up for aHUS revealed a heterozygous pathogenic variant in the complement factor H *(CFH*) gene. In addition, the complement component 3 (C3) GGTA haplotype, which has been associated with dense deposit disease, was identified. The patient also carried the *CFH* H3 risk haplotype (TGTGT), associated with a less favorable disease course and an increased risk of kidney allograft loss, as well as one copy of the aHUS risk haplotype (GGAAC) in the *CD46* gene, which encodes membrane cofactor protein (MCP). Genetic analysis also demonstrated heterozygous variants in ADAM metallopeptidase with thrombospondin type 1 motif 13 (ADAMTS13) associated with familial thrombotic thrombocytopenic purpura.

In the second patient, genetic testing revealed heterozygous *CD46* variants c.946+23G>T and c.*783T>C. The role of the c.946+23G>T variant in modulating disease risk remains uncertain; however, its presence has been associated with alternative splicing of the *CD46* transcript, involving selective inclusion of exon 8 and resulting in impaired functionality of the gene product. The c.*783T>Cvariant has been described as a risk variant for hemolytic uremic syndrome. In addition, the patient carried the CFH H3 risk haplotype (TGTGT), associated with a less favorable disease course and an increased risk of kidney allograft loss.

In both patients, eculizumab therapy was continued after kidney transplantation and was subsequently converted to ravulizumab. No recurrence of aHUS was observed after transplantation in either patient. Both patients received basiliximab induction and standard triple maintenance immunosuppression consisting of corticosteroids, mycophenolate mofetil, and tacrolimus.

All recipients underwent their first kidney transplantation in the Department and Clinic of Nephrology, Transplant Medicine and Internal Medicine at Wroclaw Medical University between April and December 2022. Protocol biopsies were not performed systematically as part of this study. Recipients without aHUS were used as the clinically relevant comparison group because healthy individuals do not carry a donor-derived allograft and therefore do not provide an appropriate biological reference for dd-cfDNA measurements. The objective was to compare early post-transplant dd-cfDNA patterns in kidney transplant recipients with and without aHUS rather than to establish reference values for total circulating cfDNA in the general population.

Blood samples were collected at four time points: one week after transplantation (D7), two weeks after transplantation (D14), one month after transplantation (M1), and two months after transplantation (M2). Missing dd-cfDNA measurements were primarily attributable to unavailable blood samples. Samples were collected into tubes dedicated to cfDNA stabilization (Cell-Free DNA BCT, blood collection tube; RUO, research use only; Streck, La Vista, NE, USA). cfDNA was isolated from 4 mL of plasma using the MagMAX Cell-Free DNA Isolation Kit (Applied Biosystems, Thermo Fisher Scientific Inc., Waltham, MA, USA), according to the manufacturer’s protocol.

cfDNA concentration was measured using fluorescence-based methods with the Quantus Fluorometer (Promega Corporation, Madison, WI, USA; Research Resource Identifier (RRID):SCR_026279) and QuantiFluor ONE double-stranded DNA (dsDNA)System (Promega Corporation, Madison, WI, USA). cfDNA quality was verified using automated electrophoresis with the 4200 TapeStation System (Agilent, Santa Clara, CA, USA; RRID:SCR_018435) and High Sensitivity D1000 ScreenTape (Agilent, Santa Clara, CA, USA).

dd-cfDNA in kidney transplant recipients was evaluated by next-generation sequencing using the AlloSeq cfDNA Kit (CareDx Inc., Brisbane, CA, USA). Sequencing was performed on a MiSeq instrument using the MiSeq Reagent v3 150-cycle Kit (Illumina Inc., San Diego, CA, USA). Sequencing results were analyzed with AlloSeq cfDNA Software (CareDx, version 2.2.0).

Donor-specific anti-HLA antibody profiling was performed using LABScreen Single Antigen Beads (One Lambda, Inc., Thermo Fisher Scientific, Canoga Park, CA, USA) according to the manufacturer’s instructions. Samples were analyzed on a Luminex FlexMAP 3D platform (Luminex Corporation, Austin, TX, USA), and results were interpreted using HLA Fusion Software, version 4.7 (One Lambda, Inc.).

Donor-specific anti-HLA antibody positivity was defined as the presence of at least one anti-HLA class I and/or class II antibody directed against the corresponding donor HLA antigen. Anti-HLA antibody specificity testing was performed only in samples with a positive screening result for anti-HLA antibodies of the respective HLA class. The final classification of antibody specificity was based on the mean normalized fluorescence intensity value (MFI_norm) obtained for bead populations corresponding to a given serological specificity with a positive or borderline signal.

For anti-HLA class I antibodies, the laboratory-defined cut-off value was MFI_norm = 1100, with a reported measurement uncertainty of 55%. Results were classified as positive when MFI_norm was ≥1700, borderline when MFI_norm was ≥500 and <1700, and negative when MFI_norm was <500. For anti-HLA class II antibodies, the laboratory-defined cut-off value was MFI_norm = 1250, with a reported measurement uncertainty of 40%. Results were classified as positive when MFI_norm was ≥1750, borderline when MFI_norm was ≥750 and <1750, and negative when MFI_norm was <750. The cut-off values were established with reference to a non-immunized male population, defined as individuals without a history of transplantation or blood transfusion.

For the purposes of the present analysis, patients were considered DSA-positive when at least one donor-specific anti-HLA antibody met the laboratory criteria for a positive result.

Descriptive analyses were conducted using STATISTICA version 13 (TIBCO Software Inc., 2017, Dell, OK, USA; RRID:SCR_014213). Given the small sample size and exploratory nature of the study, analyses were primarily descriptive. Individual trajectories are presented for the two recipients with aHUS. For recipients without aHUS, dd-cfDNA values are summarized as medians with the full observed range. Exploratory correlation analyses were performed in R version 4.1. Spearman rank correlation was used to assess the association between %dd-cfDNA and serum creatinine across the 49 available paired observations at D7, D14, M1, and M2. A partial Kendall correlation controlling for sampling time was additionally calculated using the ppcor package, version 1.1. All tests were two-sided with alpha = 0.05. Because repeated measurements were obtained within recipients and the cohort was small, these analyses were treated as exploratory and were not used to establish independence between the biomarkers. No formal a priori sample-size calculation was performed because the study was retrospective and exploratory and the aHUS subgroup was constrained by disease rarity.

The study protocol was reviewed and approved by the Bioethical Committee of Wroclaw Medical University (approval no. 37/2022, approved on 26 January 2022). All participants received and signed a written informed consent form before inclusion in the study, in accordance with the Declaration of Helsinki [24].

An artificial intelligence (AI)-based language model (GhatGPT-5.6 Thinking, OpenAI, San Francisco, CA, USA) was used to assist in language refinement. No AI tool was used for data analysis or generation of scientific conclusions.

The authors made and verified all scientific content, interpretations, and conclusions.

## 5. Conclusions

In this preliminary observational study, the two kidney transplant recipients with aHUS had early post-transplant dd-cfDNA values near or above 1% through M1, followed by a decline at M2, whereas recipients without aHUS showed a progressive decline over the early post-transplant period. Given the limited and unbalanced sample, these recipient-level observations do not establish a group-level difference or confirm that aHUS represents a biologically distinct setting. Moreover, because protocol biopsies were not systematically performed, subclinical rejection could not be excluded. The findings nevertheless raise the hypothesis that universal dd-cfDNA thresholds may require cautious interpretation in kidney transplant recipients with aHUS. Larger biopsy-anchored studies integrating dd-cfDNA with DSA, complement activation markers, endothelial injury biomarkers, and molecular graft assessment are needed to clarify the clinical significance of these observations.

## Figures and Tables

**Table 1:** Individual demographic and peri-transplant characteristics of kidney transplant recipients with and without aHUS.

Recipient	Group	WIT (min)	CIT (min)	Post-Tx Dialysis (Days)	DGF	Maintenance Immunosuppression	Induction	PRA Max (%)	Age (Years)	Sex
Non-aHUS recipients (n = 12)										
N1	non-aHUS	20	1320	0	No	S/T/MMF	ATG	83	54	F
N2	non-aHUS	15	120	0	No	S/T/MMF	Basiliximab	NA	52	F
N3	non-aHUS	30	860	0	No	S/T/MMF	None	0	69	M
N4	non-aHUS	30	1380	2	Yes	S/T/MMF	Basiliximab	20	38	F
N5	non-aHUS	34	120	0	No	S/T/MMF	Basiliximab	0	48	M
N6	non-aHUS	20	1327	0	No	S/T/MMF	Basiliximab	7	47	M
N7	non-aHUS	23	455	0	No	S/T/MMF	Basiliximab	3	73	F
N8	non-aHUS	20	120	0	No	S/T/MMF	Basiliximab	NA	33	F
N9	non-aHUS	19	129	0	No	S/T/MMF	Basiliximab	NA	32	M
N10	non-aHUS	23	855	0	No	S/T/MMF	Basiliximab	0	58	F
N11	non-aHUS	11	1465	0	No	S/T/MMF	None	0	43	M
N12	non-aHUS	19	455	0	No	S/T/MMF	Basiliximab	30	44	M
aHUS recipients (n = 2)										
A1	aHUS	27	370	0	No	S/T/MMF	Basiliximab	8	39	F
A2	aHUS	20	1680	11	Yes	S/T/MMF	Basiliximab	0	22	F

Abbreviations: aHUS, atypical hemolytic uremic syndrome; ATG, anti-thymocyte globulin; CIT, cold ischemia time; DGF, delayed graft function; F, female; M, male; min, minutes; MMF, mycophenolate mofetil; n, number of recipeints; NA, not avaiable; PRA, panel-reactive antibody; S, steroids; T, tacrolimus; Tx, transplantation; WIT, warm ischemia time. PRA max is shown as the maximum panel-reactive antibody value recorded for each recipient.

## Data Availability

The data presented in this study are available on reasonable request from the corresponding author due to privacy and ethical restrictions.
